# Genome-Wide Identification and Characterization of the *TLP* Gene Family in *Phyllostachys edulis* and Association with Witches’ Broom Disease Resistance in Bamboo

**DOI:** 10.3390/ijms241210257

**Published:** 2023-06-17

**Authors:** Yu Gu, Haoyue Yu, Sainan He, Pan Zhang, Xiaoping Ma

**Affiliations:** 1 College of Life Sciences, Sichuan Agricultural University, Chengdu 611130, China; guyu632@sicau.edu.cn (Y.G.); yuhaoyue@stu.sicau.edu.cn (H.Y.); hesainan@stu.sicau.edu.cn (S.H.); 2020316054@stu.sicau.edu.cn (P.Z.); 2 Key Laboratory of Animal Disease and Human Health of Sichuan Province, College of Veterinary Medicine, Sichuan Agricultural University, Chengdu 611130, China

**Keywords:** *Phyllostachys edulis*, thaumatin-like proteins, gene structure, gene expression, witches’ broom disease

## Abstract

Thaumatin-like proteins (TLPs) are pathogenesis-related proteins with pivotal roles in plant defense mechanisms. In this study, various bioinformatics and RNA-seq methods were used to analyze the biotic and abiotic stress responses of the TLP family in *Phyllostachys edulis*. Overall, 81 *TLP* genes were identified in *P. edulis*; 166 *TLPs* from four plant species were divided into three groups and ten subclasses, with genetic covariance observed between these species. Subcellular localization in silico studies indicated that TLPs were primarily distributed in the extracellular. Analysis of the upstream sequences of *TLPs* demonstrated the presence of cis-acting elements related to disease defense, environmental stress, and hormonal responses. Multiple sequence alignment demonstrated that most *TLPs* possessed five conserved REDDD amino acid sequences with only a few amino acid residue differences. RNA-seq analysis of *P. edulis* responses to *Aciculosporium take*, the pathogenic fungus that causes witches’ broom disease, showed that *P. edulis TLPs* (*PeTLPs*) were expressed in different organs, with the highest expression in buds. *PeTLPs* responded to both abscisic acid and salicylic acid stress. These *PeTLP* expression patterns were consistent with their gene and protein structures. Collectively, our findings provide a basis for further comprehensive analyses of the genes related to witches’ broom in *P. edulis*.

## 1. Introduction

China has the highest distribution of bamboo and is rich in bamboo forest resources, which are vital assets for advancing the country’s forestry economy. *Phyllostachys edulis* belongs to the Poaceae bamboo subfamily and is a crucial bamboo species with considerably high economic value [1].

Plants use complex mechanisms to protect themselves from pathogens. Thirteen classes of antifungal proteins have been identified based on their functional activities and structural characteristics [2]. Pathogenesis-related (PR) genes are key elements of these mechanisms. PRs are activated in response to pathogenic infections, and regulate the production of several proteins, polypeptides, and compounds that are toxic to pathogens [3]. PR proteins comprise several families with differences in their expression and biological functions. They are primarily divided into 17 groups according to their characteristics [4]. The thaumatin-like protein (TLP) family, also known as the PR protein family 5 (PR5), is a PR family whose expression is induced by certain stresses, such as pest- and disease-dependent stresses [5]. TLPs have been discovered in various organisms including nematodes, insects, fungi, gymnosperms, and angiosperms. Additionally, the typical TLP family protein sequence has a well-defined acidic cleavage domain that includes five conserved amino acids, arginine, glutamic acid (Glu), and three aspartic acid (Asp) residues (REDDD), which are thought to be involved in specific receptor binding and antifungal activities [6]. TLPs can also be activated by bacterial pathogens, plant hormones, and abiotic stresses [7]. BolTLP1 belongs to a group of TLPs that confer tolerance to salt and drought stress in broccoli and exhibit inducible expression under abiotic stress [8]. Furthermore, TLP studies in *Arabidopsis thaliana* demonstrated that overexpression of *BolTLP1* enhances tolerance to drought stress [9]. TLPs belong to the allergen family of pollens and plants [10].

Studies on the biological function of TLPs in various plants have revealed their roles in defense against fungal pathogens. The ClTLP27 TLP was identified in watermelon that significantly inhibits the growth of various fungal pathogens, including *Fusarium oxysporum* [11]. Alternatively, the *PnTLP2* gene isolated from *Panax notoginseng* is induced by *Alternaria panax* infection; the corresponding recombinant PnTLP2 protein has antifungal and defense effects [12]. A study on *Populus tremula TLP* (*PtTLP*) revealed that although *PtTLP* itself had no effect on pathogenic fungal strains, transgenic *P. tremula* with high *PtTLP* expression showed enhanced resistance to spot disease, and the leaf protein of the *PtTLP* overexpression model exhibited an apparent inhibitory effect on fungal growth in vitro [13]. Five TLPs (TLP-1, -2, -3, -4, and -5) with antifungal activities were isolated from *Medicago truncatula var. truncatula putative*. All these proteins exhibited robust antifungal activities against *Rhizoctonia solani*, *Alternaria alternata*, *Fusarium graminearum*, *Fusarium solani*, *Verticillium sp*., and *Phytophthora spp.* [14]. The mechanism underlying the antifungal activity of TLPs involves the expression of β-1,3-glucanase, which interacts with and destroys pathogenic fungal cell walls [15].

Witches’ broom disease caused by *Aciculosporium take* is highly prevalent in bamboo [16]. In bamboo infected by *A. take*, apical bud growth is inhibited and early lateral bud germination into branchlets is stimulated, resulting in several lateral branches or axillary buds that form broom-like clusters that affect bamboo growth and even cause bamboo forest decline. Therefore, bamboo TLPs in the PR protein family that have antifungal activity must be systematically analyzed to effectively control witches’ broom in bamboo.

Although *TLP* genes have been identified and functionally analyzed in many plants, no such studies have been conducted in bamboo. As the whole genome of *P. edulis* was parsed, we used bioinformatics methods to identify *TLP* genes in *P. edulis* and analyzed gene structures, upstream cis-acting elements, chromosomal locations, conserved motifs, interspecific and intraspecific phylogenetic evolution, the three-dimensional (3D) structure of the encoded proteins, gene duplication, and transcriptional expression of these *P. edulis TLPs* (*PeTLPs*). This study aimed to comprehensively understand *PeTLPs* and explore their interactions with *P. edulis* and fungal invasion, thereby providing a basis for future research.

## 2. Results

### 2.1. Identification and Protein Characteristics of PeTLPs

Eighty-one *TLP* genes were identified from the *P. edulis* genome database. The expression levels of these genes are listed in Table 1. The TLP sequences ranged from 80–650 base pair. The corresponding molecular weights of these TLPs ranged from 8598.6 Da (*PH02Gene08982*) to 71,573.6 Da (*PH02Gene13259*). The isoelectric point values of these PeTLPs ranged from 3.89–8.68 and included 28 basic proteins and 53 acidic proteins. Subcellular prediction results showed that most PeTLPs were determined to be extracellular. *PH02Gene35939*, *PH02Gene13257*, and *PH02Gene13259* were located in the plasma membrane, *PH02Gene11792* in the nucleus, and *PH02Gene11793* in the chloroplast.

### 2.2. P. edulis Multiple Sequence Alignment

**Figure 1:**
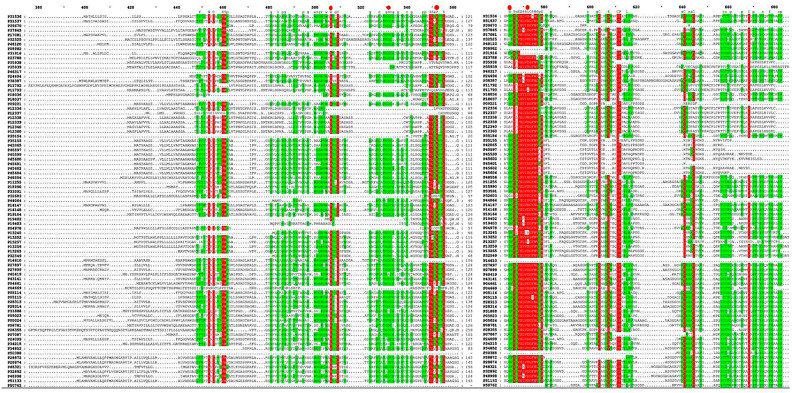
Protein sequence alignment of all identified *P. edulis* thaumatin-like proteins (PeTLPs). Conserved positions of five amino acids REDDD are labeled with a red dot. The red area is >80% similar and the green area is 50% similar. The gene name numbers correspond to the gene names identified in this study.

The multiple sequence alignment results for *P. edulis* showed high conservation among all 81 PeTLP sequences (Figure 1). Specifically, most PeTLPs contained 14 conserved cysteine residues. Most of the identified PeTLPs possessed the typical REDDD amino acid sequence.

### 2.3. Phylogenetic Analysis, Motifs, and Gene Structure

**Figure 2:**
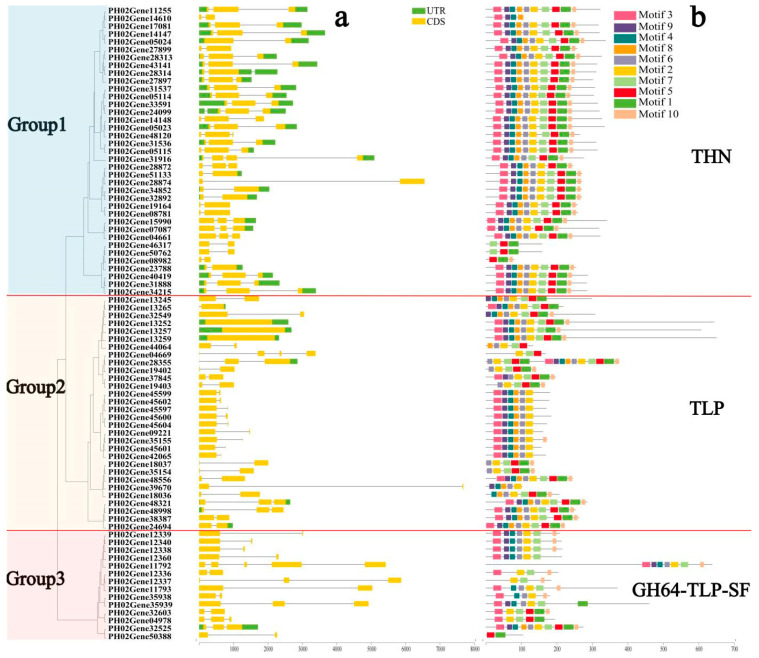
Phylogenetic tree, gene structures, and conserved motifs of PeTLPs. (**a**) Phylogenetic tree and gene structures. CDS is indicated by yellow boxes, UTR regions are indicated by green boxes, and the intron is indicated by a black line. The scale at the bottom is in base pair (bp). (**b**) Motif analysis of PeTLPs. The length of the solid line represents the length of the protein sequences. Colored boxes represent different motifs.

To study the structural diversity of PeTLPs, their exon–intron structures and motif distributions were analyzed. A corresponding phylogenetic tree was constructed using previously identified PeTLP protein sequences, and its evolutionary relationships were evaluated. As shown in Figure 2a, the 81 PeTLPs were divided into three groups (groups 1–3) according to their gene structures and functional characteristics of motifs. Group 1 contained a maximum of 37 members, which was nearly half the total number of genes. In group 1, each gene possessed one intron and at least two coding sequence (CDS) regions. Group 2 genes contained introns and CDS regions. Additionally, most group 2 genes possessed one CDS; however, three *PeTLP* genes (*PH02Gene13252*, *PH02Gene13257*, *PH02Gene13259*) contained no introns. Group 3 comprised the smallest PeTLP group, with 14 members. In group 3, only one gene (*PH02Gene32525*) possessed two untranslated regions (UTRs), whereas the remaining genes had no UTRs.

The conserved regions of proteins form the basis of their function. To further reveal the structural diversity and functional characteristics of the PeTLPs, their motif patterns were investigated (Figure 2b). Thaumatin (THN), TLP, and GH64-TLP-SF represent group 1, 2, and 3 TLPs, respectively. Motifs 2 and 3 were detected in most *TLP* gene sequences. The motifs differed between groups; however, members within the same group tended to exhibit similar motif patterns. For example, in group 1 (THN), motif 5 was identified as a characteristic motif. Motif 2 was a characteristic structure of group 2 (TLP). The group 3 (GH64-TLP-SF) superfamily possessed motif 7, except for *PH02Gene50388*. These results confirmed that the gene structure and motif patterns influenced phylogenetic diversity.

### 2.4. Evolutionary Associations between Species

**Figure 3:**
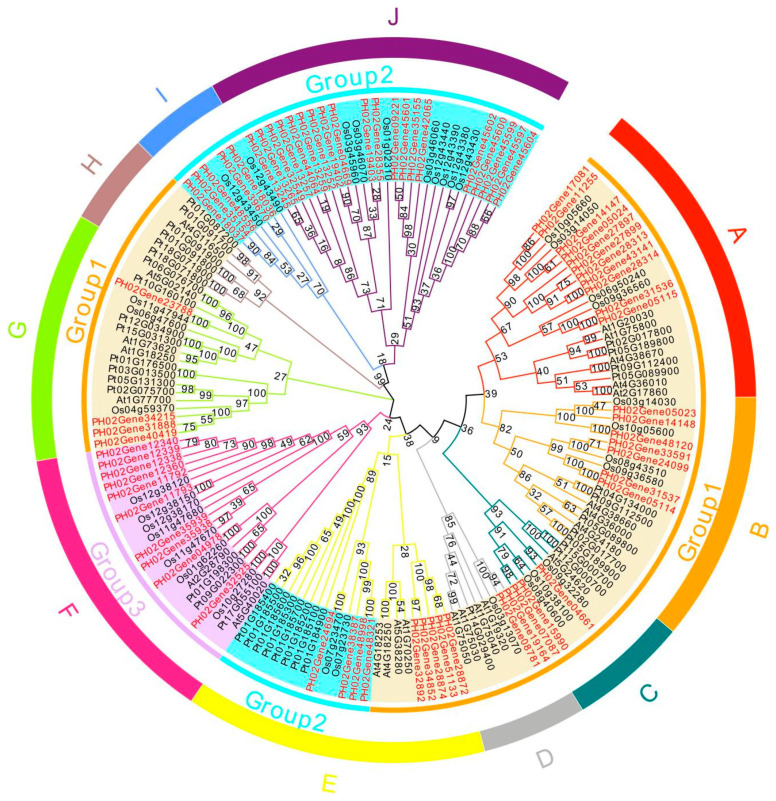
Phylogenetic analysis of TLPs. At: *Arabidopsis thaliana*; Os: *Oryza sativa*; Pt: *Populus tremula*. Different colored branches indicate different subclasses (A, B, C, D, E, F, G, H, I, and J). Genes in red font indicate the *PeTLPs*. Numbers on the phylogenetic tree represent confidence levels.

To explore the evolutionary relationships of the TLP family among different species, we collected 166 TLPs from four plant species (*P. edulis*, *A. thaliana*, *Oryza sativa*, and *P. tremula*) and constructed phylogenetic trees (Figure 3). The corresponding tree results were divided into three large groups and ten subclasses. According to the evolutionary analysis, the *TLP* gene families of the four species can be divided into ten subclasses (A, B, C, D, E, F, G, H, I, and J) to more precisely demonstrate evolutionary and functional relationships of *TLP* genes between *P. edulis* and other species.

We divided PeTLPs into three groups based on their internal evolutionary relationships (Figure 2a). The constructed evolutionary tree of *TLP* gene families from different species revealed that group 1 and group 2 *PeTLP* members exhibited changes in their evolutionary relationships and showed a scattered distribution. Group 1 was divided into six subclasses (A, B, C, D, I, and J) and group 2 into three subclasses (E, I, and J) (Figure 3).

Multiple pairs of homologous genes were found among the *P. edulis*, *A. thaliana*, *O. sativa*, and *P. tremula TLPs*. The largest TLP subclass was J, which contained 28 *TLPs*, and the smallest was I, which contained only seven members. In addition to subclass H, nine other subclasses comprised TLPs from both *P. edulis* and other plant species, indicating a close relationship between *PeTLPs* and *TLPs* from other species. Among the subclasses, there were homologous relationships between *P. edulis*, *A. thaliana*, *O. sativa*, and *P. tremula* in six subclasses (A, B, C, E, F, and G). Overall, *PeTLPs* were primarily distributed in subclasses I, J, and F; however, subclasses I and J only exhibited homology between *P. edulis* and *O. sativa*, which were the two species that have been identified to have the highest homology.

### 2.5. Distribution of PeTLPs on Scaffolds

**Figure 4:**
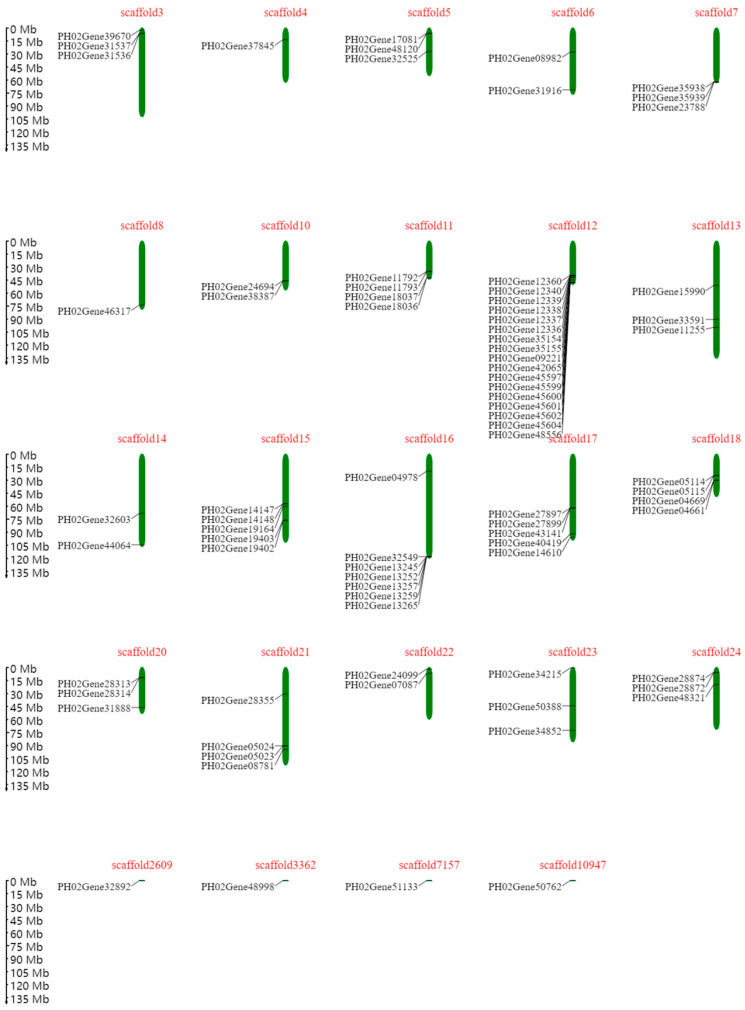
Distribution of *PeTLP* genes on scaffolds in *P. edulis*. Scaffolds are represented by green bar boxes, and the different horizontal lines on the scaffold represent the genes at different locations. The scaffold numbers are indicated at the top of the scaffolds. The scale on the left is in megabases (Mb).

Based on their chromosomal location, *PeTLPs* were unevenly distributed on *P. edulis* scaffolds (Figure 4). Scaffold 12 contained the most *PeTLPs*, with 17 genes, followed by scaffold 16. However, only one *PeTLP* was present on scaffolds 4, 8, 2609, 3363, 7157, and 10,947.

### 2.6. Interspecific and Intraspecific Gene Collinearity Analysis

**Figure 5:**
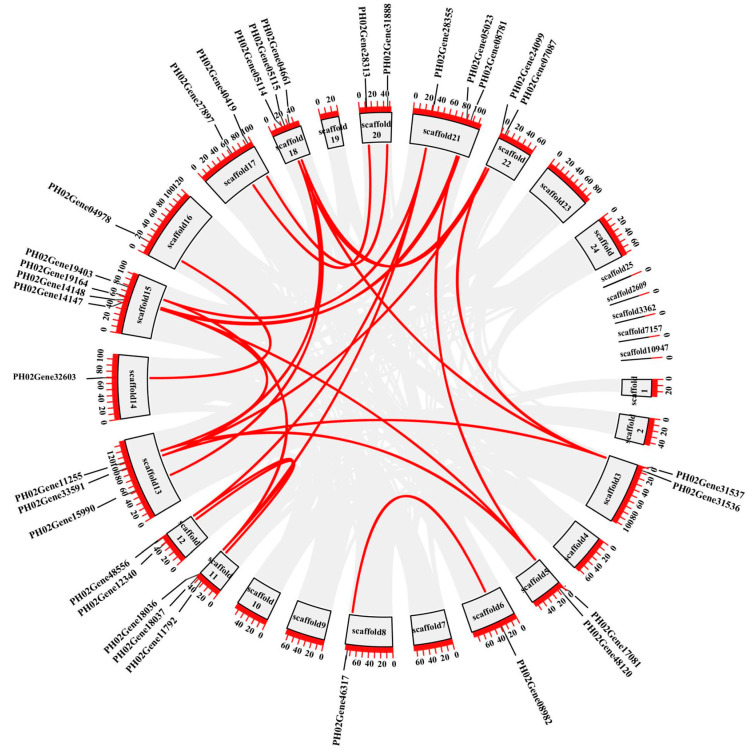
Collinearity analysis of PeTLPs in *P. edulis*. The gray line segments indicate all collinearity relationships, and the red line segments indicate collinearity relationships of PeTLPs. The black boxes on the outside represent different scaffolds.

The results of the intraspecific collinearity analysis are shown in Figure 5. In this analysis, a total of 26 collinear gene pairs were obtained. Most collinear relationships occurred on scaffolds with a larger number of genes. Scaffolds 15 and 21 had the largest number of collinear gene pairs, whereas scaffolds 1, 2, 4, 7, 9, 10, 19, 23, 24, and 25 had no collinear gene pairs. These findings suggested that the large-scale duplication of chromosomal segments may have caused the expansion of the PeTLP gene family.

**Figure 6:**
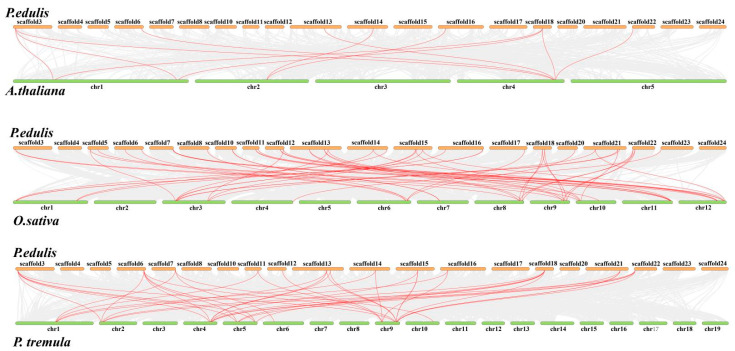
Comparative linear relationship of TLPs in *P. edulis*, *A. thaliana*, *O. sativa*, and *P. tremula*.

Comparative collinear maps of three representative plants were constructed at a genome level, including one monocotyledon (*O. sativa*), one dicotyledonous herb (*A. thaliana*), and one dicotyledonous woody plant (*P. tremula*), as shown in Figure 6. A total of 47 *PeTLP* genes exhibited syntenic relationships with those in *O. sativa*. The numbers of *PeTLP* genes with syntenic relationships with *P. tremula* and *A. thaliana* were 35 and 11, respectively. Scaffold 3 (*PH03Gene31537*), scaffold 6 (*PH03Gene08982*), scaffold 13 (*PH02Gene11255*), and scaffold 14 (*PH02Gene32603*) were homologous to all three species (Figure 5 and Figure 6).

Gray lines in the background represent the synteny blocks of *P. edulis* and other plants, while the red lines highlight the collinearity of TLP gene pairs.

### 2.7. Detection of cis-Regulatory Elements in PeTLPs

**Figure 7:**
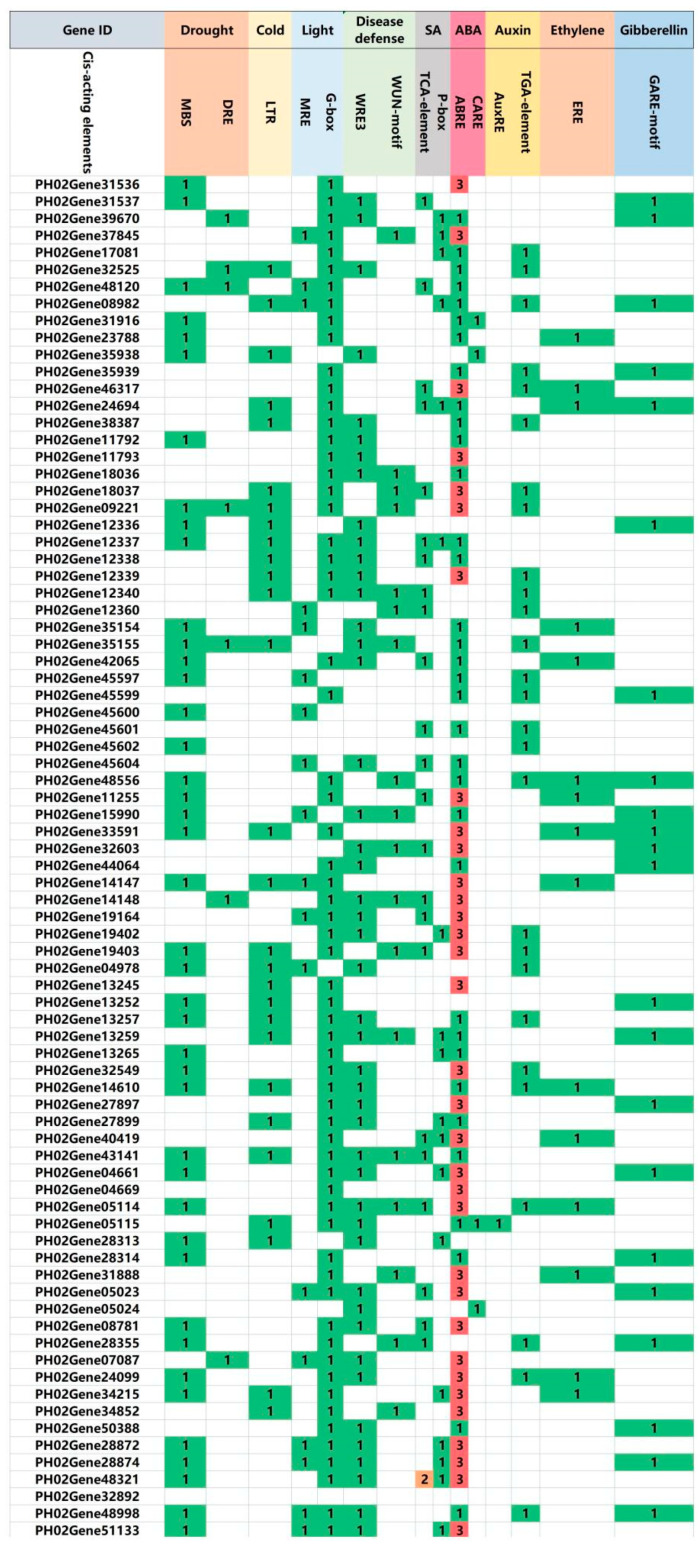
Prediction of cis-acting elements in *PeTLP* promoter regions. The cis elements are indicated by different colored boxes with numbers.

Cis-acting elements play crucial important roles in gene expression and reveal gene function. As shown in Figure S1, 15 representative cis-acting elements were selected. These included seven stress response elements (e.g., drought, cold, disease defense, and light) and eight hormone response elements, such as abscisic acid (ABA), salicylic acid (SA), auxin, gibberellin, and ethylene. As shown in Figure 7, two cis-acting elements were determined to be related to disease defense: WRE3 (51 genes) and the WUN motif (20 genes). Additionally, WUN and WRE3 were detected in 10 genes (*PH02Gene05114*, *PH02Gene09221*, *PH02Gene12340*, *PH02Gene13259*, *PH02Gene14148*, *PH02Gene15990*, *PH02Gene18036*, *PH02Gene19403*, *PH02Gene35155*, and *PH02Gene43141*). Hormone-related elements were present in a large proportion of the *PeTLPs* (n = 79). Among the ABA-responsive elements, ABA response elements (ABRE) were present in many genes. Thirty-three genes contained three ABRE elements. *PH02Gene05114*, *PH02Gene05115*, *PH02Gene08982*, *PH02Gene34215*, *PH02Gene40419*, *PH02Gene46317*, *PH02Gene48556*, and *PH02Gene50762* contained four hormone-related elements. Overall, 75 of these genes contained both hormonal and stress response elements. The findings indicate that PeTLPs may play an important role in forming broom-like branches and contribute to the corresponding resistance of *P. edulis* to witches’ broom.

### 2.8. Transcription and Expression of PeTLPs following Pathogen Infection

TLPs belong to the PR protein family, which primarily contribute to pathogen resistance. The response of bamboo to pathogens at the transcriptome level has not yet been reported. Thus, the transcript profiles of *P. edulis* in response to *A. take* infection were determined using ribonucleic acid sequencing (RNA-seq) technology. Overall, messenger ribonucleic acid expression in the apical buds, lateral buds, leaves, and stems of *P. edulis* was detected after infection with *A. take*. Healthy tissues were used as controls. A |Log2 fold-change (Log2FC)| > 1 and false discovery rate < 0.05 were the criteria to identify differentially expressed genes (DEGs).

**Figure 8:**
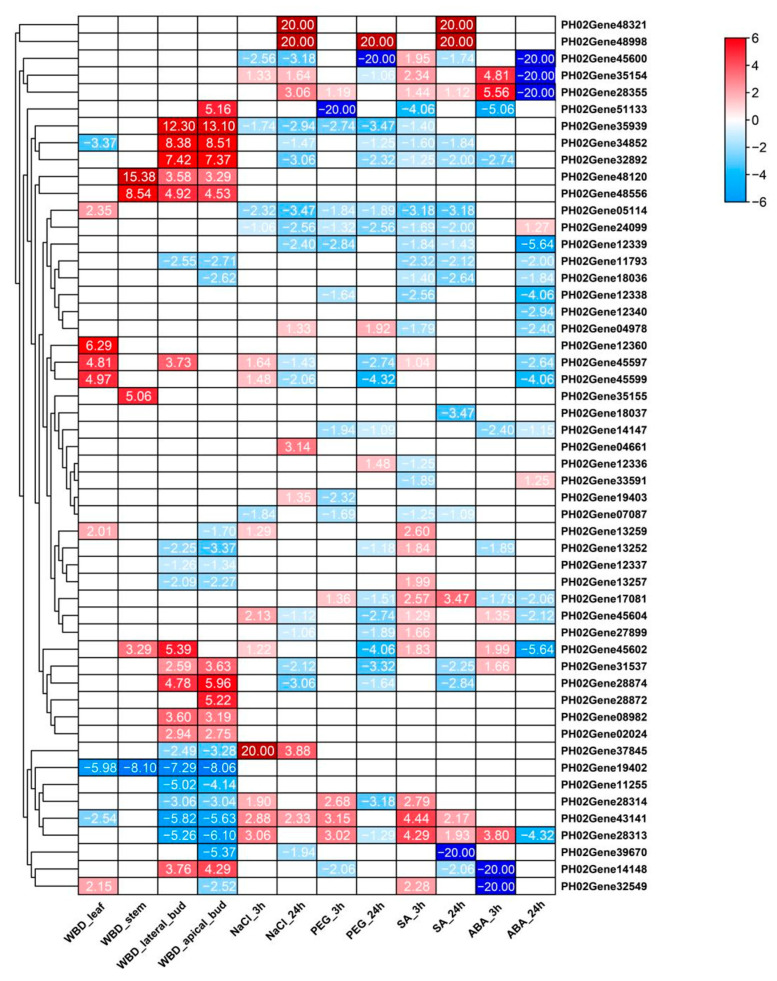
Heatmap of transcriptional expression levels of *PeTLPs* in *P. edulis* witches’ broom disease (WBD) within different tissues, and the corresponding response to NaCl, polyethylene glycol (PEG), salicylic acid (SA), and abscisic acid (ABA) stress, expressed as Log2FC values. Blocks with colors indicate the level of upregulation (red) or downregulation (blue). White blocks indicate genes that are not differentially expressed.

As shown in Figure 8, 12 DEGs were upregulated in both apical and lateral buds, whereas 14 and 10 were downregulated in the apical and lateral buds, respectively. Additionally, six and four DEGs were upregulated in leaves and three and one DEGs were downregulated in stems. *A. take* predominantly infect bamboo buds, thereby inducing these buds to continuously differentiate to form witches’ brooms. However, this pathogen had minimal interaction with stems and leaves. The results of the present study are consistent with this phenomenon.

The relative expression levels of *PH02Gene34852*, *PH02Gene32892*, *PH02Gene35939*, *PH02Gene48556*, *PH02Gene28874*, and *PH02Gene28872* were more than four times higher in buds infected with *A. take*. *PH02Gene48556* and *PH02Gene48120* were notably upregulated in both buds and stems. Five genes (*PH02Gene11255*, *PH02Gene28313*, *PH02Gene43141*, *PH02Gene19402*, and *PH02Gene31888*) were notably downregulated (four times) in infected *A. take*. In particular, *PH02Gene19402* was strongly downregulated in all four plant tissues. These findings indicated that these genes play crucial roles in the response of *P. edulis* to *A. take* infection.

### 2.9. Analysis of PeTLPs Gene Expression in Witches’ Broom Disease Buds via qRT-PCR

**Figure 9:**
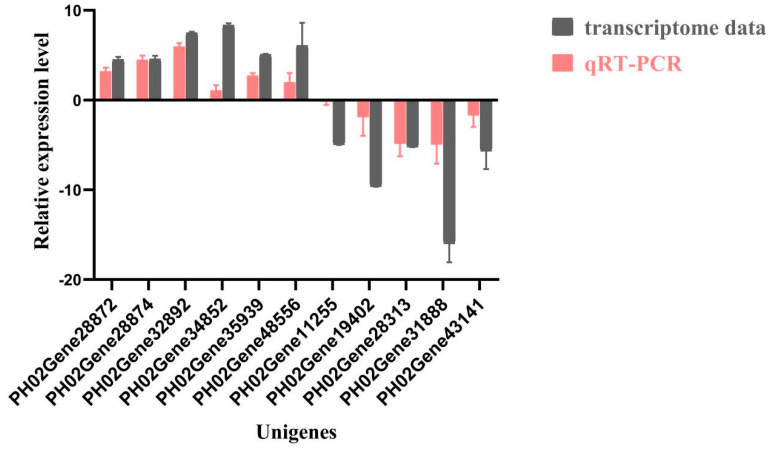
Gene expression of identified *PeTLPs* in the buds after fungal infection. The *X*-axis indicates the names of the 11 DEGs in bamboo buds, while the *Y*-axis shows the gene expression level. These values are represented by mean ± SDs, and the line in the bar indicates standard deviations. Brown bars represent transcriptome results, and red bars indicate qRT-PCR results.

To further investigate the differences in *PeTLP* expression levels in *P. edulis* buds after *A. take* infection, qRT-PCR was performed to verify the expression of 11 transcripts with notably different expression levels (Figure 9). Compared with healthy shoots, the relative expression levels of *PH02Gene34852*, *PH02Gene32892*, *PH02Gene35939*, *PH02Gene48556*, *PH02Gene28874*, and *PH02Gene28872* were notably higher in diseased bamboo shoots, with the highest expression level observed for *PH02Gene32892* (5.96× higher than that in healthy shoots). Contrastingly, the expression levels of *PH02Gene11255*, *PH02Gene28313*, *PH02Gene43141*, *PH02Gene19402*, and *PH02Gene31888* were notably lower in the diseased bamboo shoots. In particular, the expression level of *PH02Gene31888* was 4.97× less than that in healthy shoots. Overall, the qRT-PCR results were consistent with the transcriptome data. The comparative statistical analysis results of the gene expression acquired by qRT-PCR and RNA-Seq in healthy and diseased buds were shown in Figure S2.

### 2.10. Expression Profiles of PeTLPs in Response to Abiotic Stress

Salt and drought stress treatments were performed at 3 and 24 h using NaCl and polyethylene glycol (PEG), respectively. The leaf *PeTLPs* expression profile of the transcriptome is shown as a heatmap (Figure 8).

Under NaCl treatment, ten and nine DEGs were upregulated, and five and fourteen DEGs were downregulated at 3 and 24 h, respectively. In the PEG treatment group, five and three DEGs were upregulated, and ten and nineteen DEGs were downregulated at 3 h and 24 h, respectively. As the duration of abiotic stress increased, the number of *PeTLPs* that mediated the corresponding stress response increased along with an increase in the function of these *PeTLPs*.

### 2.11. Expression Profiles of PeTLPs in Response to Phytohormones

Promoter analysis demonstrated that most *PeTLPs* contained multiple hormone stress response sites. Therefore, we used the transcriptome data of different genes to verify whether their expression levels were induced by SA and ABA treatments (Figure 8).

Under ABA treatment, six and seven DEGs were notably upregulated and downregulated, respectively, at 3 h. *PH02Gene28355* displayed the highest expression, followed by *PH02Gene35154*. In contrast, at 24 h, only two DEGs were upregulated and sixteen DEGs were downregulated. Most DEGs that were upregulated at 3 h were downregulated at 24 h. Additionally, *PH02Gene45602* and *PH02Gene12339* were notably downregulated.

Next, we evaluated the effects of SA treatment on plant disease resistance. Under SA treatment, fifteen and six DEGs were upregulated at 3 h and fourteen DEGs were downregulated at 24 h. DEGs at 3 h showed almost identical expression patterns to those at 24 h. The findings suggested that pathogen resistance of *PeTLPs* was mediated by SA.

### 2.12. Consistency of Expression Pattern and Structure of PeTLPs

**Figure 10:**
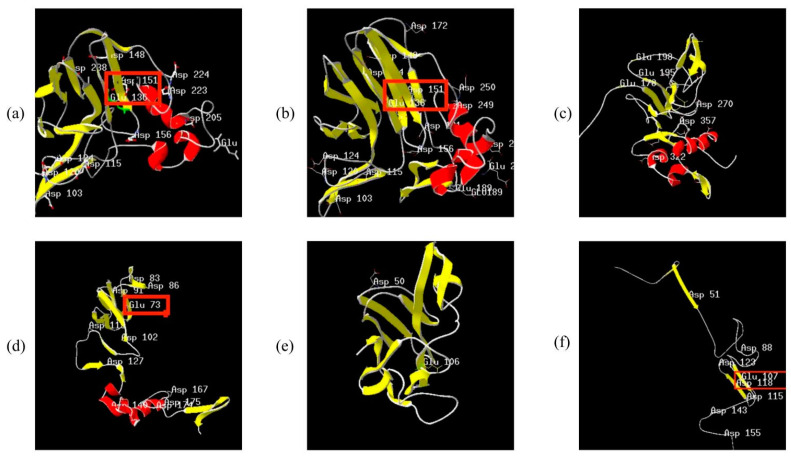
The 3D structure of TLPs in *P. edulis*. The red area indicates α-helix, yellow indicates β-sheet, and gray indicates random coil structure. (**a**) PH02Gene28872. (**b**) PH02Gene28874. (**c**) PH02Gene28355. (**d**) PH02Gene37845. (**e**) PH02Gene45599. (**f**) PH02Gene45600.

Some genes in the evolutionary tree (Figure 2) within the PeTLP family were in the same branch, which was consistent with the expression of these genes during transcriptome stress and hormone responses (Figure 8). *PH02Gene28872*, *PH02Gene51133*, *PH02Gene28874*, *PH02Gene34852*, and *PH02Gene32892* belonged to the same branch in group 1 and were notably upregulated in the lateral buds following pathogen infection. Contrastingly, none of these genes were notably upregulated or downregulated under other stresses. Representative protein sequences were selected for 3D structure prediction and the best model was determined based on the corresponding evaluation (Figure 10). The primary structures of PH02Gene28872 (Figure 10a) and PH02Gene28874 (Figure 10b) indicated that each protein contained Glu-Asp amino acid residues, with more Asp than Glu. Acidic amino acid residues were conserved in the secondary and 3D structures of edmunds. The corresponding secondary structure analysis determined that this group of proteins included α-helix, β-sheet, and random coil structures. Five conserved acidic sites (Glu136, Asp148, Asp151, Asp238, and Asp103) were present in PH02Gene28872 (Figure 10a) within the β-sheet. In contrast, a variable acidic residue (Asp205) was located under the surface of the α-helix, and the Asp151-Glu136 pair acted as a catalytic center. Interestingly, the same pair of acidic amino acids (Asp151-Glu136) was also found in PH02Gene28874 (Figure 10b) with a Glu/Asp spatial conformation within an antiparallel sheet.

In group 2, the expression patterns and structures of *PH02Gene28355*, *PH02Gene19402*, *PH02Gene37845*, and *PH02Gene19403* were similar. These genes were repressed in response to fungal infection and were less likely to be expressed under hormonal and abiotic stresses. Analysis of their protein structures revealed the presence of three Asp and Glu residues in PH02Gene28355 (Figure 10c). Secondary structure analysis also indicated the presence of α-helix and β-sheet; however, Asp and Glu did not occur in pairs. The same findings were evident in the structure of PH02Gene37845 (Figure 10d) with only one Glu and no paired Asp/Glu. Therefore, we speculate that no catalytically active center existed within these protein structures and this group of proteins could not respond strongly to fungal and abiotic stresses.

Notably, analysis of the protein structures of PH02Gene45599 (Figure 10e) and PH02Gene45600 (Figure 10f) revealed β-sheet structure with no α-helix structure. In these proteins, both Asp and Glu residues were located within this β-sheet. PH02Gene45599, PH02Gene45602, PH02Gene45597, PH02Gene45600, and PH02Gene45604 proteins, which belong to the same branch, exhibited the same trend in transcriptome expression patterns. All these proteins were upregulated in the disease response; additionally, their corresponding expression patterns were almost identically downregulated in the salt, drought, SA, and ABA treatments.

## 3. Discussion

TLPs have been identified in various plants. These proteins play vital roles in the defense response of plants, as evidenced by the increasing number of studies. However, TLPs vary in quantity across different plants. In this study, 81 *TLP* genes were identified in *P. edulis*. Other plants have fewer *TLP* genes, such as melons (n = 28), grapes (n = 33) [17], and western white pine (n = 10) [18].

Additionally, we mapped *PeTLPs* to their corresponding scaffolds based on the location information of the *P. edulis* genome. The *PeTLPs* were unevenly distributed on the *P. edulis* scaffolds. Because of the relatively large size of the *P. edulis* genome, its assembly is not optimal. Many studies have analyzed the evolutionary relationships of TLP families among different species. The 118 TLPs from plants, animals, and fungi have been classified into nine major groups, with five groups containing most of the plant species [6]. Here, we divided the 166 *TLP* genes from two dicotyledons and two monocotyledons into 10 subclasses (Figure 3). The distribution of TLPs was uneven across plants, suggesting that the expansion of TLPs differs among plant species. For example, only eight *TLP* genes in *O. sativa* and 20 PeTLPs were detected in the subclass with the largest number of genes, subclass J. In contrast, subclass H displayed no *TLP* genes in *O. sativa* or *P. edulis*. Interestingly, three *TLP* genes from *A. thaliana* (*At1G75800*, *At1G20030*, *At4G36010*) were found in subclass A, which contains members that reportedly participate in the ABA signaling pathway and abiotic stress responses [7]. Thus, members of subclass A may function in abiotic stress pathways. Genes in the same subclass were the closest evolutionarily related genes, with the most similar functions. There were 11 *PeTLPs* in subclass A, of which two genes were strongly differentially expressed following ABA treatment. Additionally, the functions of unknown PeTLP members can be predicted and verified based on the established functions of *TLP* gene family members from other species.

Previous studies on the phylogeny of TLPs have suggested that the family of plant TLPs is phylogenetically related and may have originated from the common ancestral genes of gymnosperms and monocots 130 million years ago [19]. Collinear analysis in the present study revealed that *O. sativa* and *P. edulis* had the most collinear genes, presumably because they both belonged to the Poaceae family. A previous study identified six tandem-replicated regions and 12 fragment-replicated gene pairs in melon [20], which is consistent with our results. These results suggested that tandem and fragment duplications are common in the TLP family and may be the main drivers of the PeTLP family expansion.

The subcellular localization results demonstrated that most of the proteins were extracellular. Corresponding relevant studies have shown that most barley TLPs are located extracellularly, indicating that they may be involved in seed germination [21]. However, *Camellia sinensis* TLPs are primarily localized in the cell membrane and notably improve drought resistance in *Arabidopsis* [22]. *Arabidopsis* pathogenesis-related 1, a vital defense protein, has been detected in the extracellular space [23]. Protein expression is closely related to its function. Therefore, different proteins have different functions. We speculate that PeTLPs with different subcellular localizations possess different mechanisms of action.

Further analysis indicated that PeTLPs exhibit certain characteristics in terms of intron/exon patterns, motif structures, and phylogenetic relationships. A total of 26 collinear gene pairs were identified. In addition, several genes with tandem duplications were classified into the same group, such as *PH02Gene40419* and *PH02Gene31888*, which were classified in group 1, indicating that they may have originated from recent gene duplications. These results suggest that the tandem duplication of genes was the primary reason for the expansion of PeTLPs. Similar results have been reported in other studies, and exon-intron patterns in the same phylogenetic classification showed considerable similarity [24]. Thus, the present classification results were reliable.

Sequence alignment revealed that PeTLPs contain characteristic cysteine residues and highly conserved REDDD amino acids related to their antifungal activity [20]. Specifically, cysteine residues are thought to be necessary for the high stability of proteins under extreme environmental conditions, whereas REDDD sequences are thought to be involved in maintaining the proper topology and surface electrostatic potential around acid cleavage. These properties are likely critical for the antifungal ability of PeTLPs [25].

Two acidic amino acid residues, Glu and Asp, are considered nucleophiles and proton donors for glycosidic bond cleavage. Glu/Asp-Glu/Asp is the active center of TLPs, with an antiparallel sheet spatial conformation [26]. The 3D structure of the protein predicted in the present study was determined to possess a catalytically active center for Glu–Asp, and the β-sheet of these genes was highly responsive to the pathogen. These findings were consistent with the transcriptome results, which indicated that this gene was activated during infection, confirming the antifungal activity of PeTLPs. In contrast, PH02Gene28355 and PH02Gene37845 did not contain a paired Glu/Asp; therefore, these proteins could not be activated in response to the disease. Interestingly, the evolutionary tree of the PeTLP family demonstrated that some genes belonging to the same branch had the same expression trend as the branches of biotic and abiotic stress responses. Therefore, we hypothesized that structural similarity might also lead to the same functional and stress responses.

The analysis of cis-acting elements revealed that most genes contained domains related to pathogen defense, such as the WRE3 and WUN motif. The cis-acting elements of TLPs demonstrate that these proteins respond to biotic and abiotic stresses [27]. We speculated that PeTLPs play a vital role in the defense against *A. take*. This is consistent with the results of the antifungal activity in a previous protein comparison experiment. A similar set of elements was found in the promoters of *Gossypium barbadense* TLPs, and different elements in the promoter region of GbTLPs have been suggested to play pivotal roles in abiotic stress and hormonal responses [28]. *PH02Gene28874* and *PH02Gene28872* contained WRE3 cis-acting elements. Furthermore, *PH02Gene34852* and *PH02Gene48556* possessed a WUN motif, which was consistent with the transcriptional expression levels of witches’ broom disease in *P. edulis*. Simultaneously, these genes were significantly upregulated in disease response transcriptional data.

Transcriptional analysis of fungal infections showed that most *PeTLPs* were tissue-specific and upregulated (Figure 8). Additionally, more upregulated genes were observed in the buds. The expression levels of *PH02Gene34852*, *PH02Gene35939*, *PH02Gene48556*, *PH02Gene28874*, and *PH02Gene28872* were upregulated, which was consistent with the understanding that these genes contain disease response-related cis-acting elements. Previous studies have established that the occurrence of witches’ broom in plants is caused by changes in plant hormones, especially auxin, which leads to the germination of plant axillary buds and ultimately exhibits characteristic clustering symptoms [29]. These observations were consistent with the results of transcriptome sequencing in the present study, which showed that both the lateral and apical buds contained upregulated genes. Infection with *A. take* resulted in a stronger induction of the expression of *PeTLPs* in the buds than in stems and leaves. The collective findings indicated that the formation of arbuscular symptoms was primarily caused by bud infection, and pathogenic fungi predominantly existed within the buds. In contrast, little or no infection occurred in the stems and leaves.

ABA is a plant hormone which has a vital role in plant growth and development and is regulated by fungal infections in host plants [30]. ABA integrates stress signals to control stress responses and enables plants to adapt to various stress environments [31]. The analysis of cis-acting elements indicated that almost all *PeTLPs* contain ABA-responsive elements, such as ABRE and CARE promoter elements, with ABRE being the most abundant cis-acting element within these *PeTLPs*. One ABRE-responsive element was detected in *PH02Gene35154*, and *PH02Gene33591, PH02Gene37845*, *PH02Gene46317*, and *PH02Gene44064* each possessed three ABREs (Figure 7). The heatmap of the hormone response (Figure 8) demonstrated that *PH02Gene35154* was significantly upregulated after 3 h of ABA stress and *PH02Gene33591* was significantly upregulated after 24 h of ABA stress. These findings indicate that these genes could be actively involved in the ABA stress response, corresponding to the promoter analysis results. 

Fungal-mediated biotic stress activates the plant immune system by sensing pathogen-associated signals to induce SA signaling and upregulate PR genes [32]. As shown in Figure 8, the upregulated genes were notably expressed at 3 and 24 h after SA treatment. For example, *PH02Gene48321* was notably upregulated, and the corresponding cis element results indicated that this gene contained two TCA elements, which was consistent with the SA response. Overall, the expression profiles under SA and ABA stress revealed that *PeTLPs* were associated with plant disease resistance.

*TLP* genes could potentially be used as molecular markers of fungal disease resistance [33]. Therefore, our results can be used in breeding studies to improve the resistance of *P. edulis* to witches’ broom.

Numerous studies on the biological roles of TLPs have demonstrated their antifungal activity. However, details regarding their functions in new species and specific individuals remain uncertain. Therefore, further studies on PeTLPs are required to better understand the roles of these molecules in *P. edulis*.

## 4. Materials and Methods

### 4.1. Plant Materials

In this study, a 5-year-old *P. edulis* was cultivated at the experimental base of Sichuan Agricultural University, Ya’an City, Sichuan Province, China (29°58′40″ N, 103°0′13″ E). The *P. edulis* in this experimental base was partially inoculated with *A. take* two years ago and is now in the peak period of witches’ broom disease. The bamboo in which the witches’ broom symptoms appeared was used as a disease group. The apical buds of white fruiting bodies of *A. take* with obvious arbuscular symptoms, lateral buds without white fruiting bodies at the lower ends of the arbuscular branches, leaves on the arbuscular branches, and first and second stems at the end of the arbuscular branches were collected in mid-April. Bamboo tissue without arbuscular branches in whole plant was used as a control. All the treatments were conducted in triplicate, and all the samples were stored at −80 °C for RNA-seq and qRT-PCR analysis.

### 4.2. Acquisition of P. edulis Genome Information

The genome and protein sequences of *P. edulis* were downloaded from the BambooGDB database (http://www.bamboogdb.org/ (accessed on 1 September 2022)) and saved in cds, genome, gff, and pep formats.

### 4.3. Identification Analysis

To identify *TLP* genes, the Hidden Markov Model (HMM) search method was used. HMMSEARCH was used with a threshold e-value < 0.001. Thereafter, we used a species-specific HMM to search for domains, proteins, and cds sequences.

Sequences obtained from the HMM search were screened and identified according to the definition of the thaumatin domain (ID: PF00314) in the Pfam database (http://pfam.xfam.org/ (accessed on 1 September 2022)). The TLP domain sequence was confirmed using the Conserved Domain Database (CDD; https://www.ncbi.nlm.nih.gov/cdd/ (accessed on 1 September 2022)) and a reliable *P. edulis TLP* gene sequence was obtained.

### 4.4. Analyses of Protein Properties of PeTLPs

ExPASy (https://web.expasy.org/protparam/ (accessed on 10 September 2022)) [34] was used to predict the characteristics of the PeTLPs, including length, isoelectric point (pI), and molecular weight (MW). CELLO (http://cello.life.nctu.edu.tw/ (accessed on 10 September 2022)) [35] was used to predict the subcellular localization of the proteins.

### 4.5. Motif, Gene Structure, and Scaffold Location Analyses

PeTLP motifs were identified using the MEME tool (http://meme-suite.org/ (accessed on 22 September 2022)) with the default parameter settings of maximum number of motifs = 50 and minimum number of motifs = 10. The corresponding results were shown using TBtools [36]. NCBI-CDD (https://www.ncbi.nlm.nih.gov/cdd (accessed on 22 September 2022)) was used to analyze motif function.

Exon–intron structures were mapped using the Gene Structure Display Server software (http://gsds.gao-lab.org/ (accessed on 22 September 2022)) according to the *P. edulis* TLP transcript ID [37].

We used the location information file of the *PeTLP* gene on the scaffolds, the genome scaffold length file, and MapChart software for figure construction to obtain the physical location information of the genes on the scaffolds.

### 4.6. Cis-Regulatory Element Analysis

We obtained the position information of the *PeTLP* genes on the chromosome using a Linux system and then extracted the promoter information based on the location information. Cis-acting element analysis was performed using the PlantCARE database (http://bioinformatics.psb.ugent.be/webtools/plantcare/html/ (accessed on 12 October 2022)). Finally, we submitted the gene and feature beds to the GSDS website (http://gsds.cbi.pku.edu.cn/ (accessed on 12 October 2022)) to map the cis-acting elements of the genes [38].

### 4.7. Phylogenetic Tree Construction and Synteny Analysis of PeTLP Genes

ClustalW was used for multiple sequence alignment to evaluate the evolutionary relationships of the *TLP* genes in *P. edulis* using default parameters [39], and Molecular Evolutionary Genetics Analysis (MEGA) was used to construct a maximum likelihood phylogenetic tree [40]. The amino acid substitution model chosen was WAG + I + G4. The *PeTLP* collinear gene files were uploaded to TBtools software to produce the corresponding collinear circle map.

### 4.8. Multiple Sequence Alignment

PeTLP sequences were aligned using MEGA7 software. The results were edited using GeneDoc software (http://www.psc.edu/biomed/genedoc/ (accessed on 2 November 2022)).

### 4.9. Evolutionary Relationship and Collinearity Analysis of Different Species

The TLP amino acid sequences identified in three species (*A. thaliana*, *O. sativa*, and *P. tremula*) were obtained from NCBI. ClustalW 2.0.10 software was used to compare multiple amino acid sequences between PeTLPs and TLP proteins in other plant species. A phylogenetic tree was constructed using the neighbor-joining method with MEGA 7.0 software. The phylogenetic tree was edited using EvolView (https://www.evolgenius.info (accessed on 2 November 2022)).

Genome-wide replication events were obtained using the One Step MCScanX module in TBtools 1.1047 software to determine the gene replication relationships of PeTLPs. A genome-wide collinearity analysis was performed for *P. edulis* with *A. thaliana*, *O. sativa*, and *P. tremula*. The “Amazing Super Circos” package was used to visualize gene localization and linear relationships on the respective chromosomes [41].

### 4.10. Transcriptomic Data Sets to Analyze Expression Patterns of PeTLPs in Response to Phytohormones and Abiotic Stress

We obtained publicly available transcriptome data from NCBI (https://www.ncbi.nlm.nih.gov/geo/query/acc.cgi?acc=GSE169067 (accessed on 2 December 2022)) to determine the gene expression profiles of PeTLPs under different stresses. In the present study, transcriptome data were obtained from the expression levels of leaf *PeTLPs* under SA, ABA, NaCl, and PEG stress at different time points. Data were normalized to fragments per kilobase of transcript per million mapped reads. To calculate the Log2FC value of genes, clustering [42], and normalization analysis, a heatmap of the relative *PeTLPs* expression levels was generated using the Lianchuan Biological Cloud Platform (https://www.omicstudio.cn/index (accessed on 2 December 2022)).

### 4.11. Expression Pattern of PeTLPs in Pathogen Infection

Total RNA was extracted using TRIzol reagent (AG, Hunan, China) according to the manufacturer’s instructions, RNA samples with high quality were determined using an Agilent 2100 Bioanalyzer system (Santa Clara, CA, USA). The Illumina platform was used for transcriptome analysis by employing a 150-bp paired-end library according to the manufacturer’s instructions (San Diego, CA, USA). Each sample was paired-end sequenced with an Illumina HiSeq 4000 platform. Low-quality reads were filtered. The sequencing data were compared to the reference species *P. edulis*. Differential genes between samples were analyzed using the R package edgeR. A |Log2 fold-change (Log2FC)| > 1 and false discovery rate < 0.05 were the criteria to identify differentially expressed genes (DEGs). A heatmap of transcriptional expression levels of differentially significant expression *PeTLPs* in *P. edulis* with witches’ broom disease was generated by Heatmapper and expressed as Log2FC values [43].

### 4.12. Protein Structure Prediction

Gene sequences were selected using the results of transcriptome analysis to construct a 3D protein model on the SWISS-MODEL website (https://swissmodel.expasy.org/ (accessed on 12 December 2022)) [44]. The generated model was assessed using SAVES software (https://saves.mbi.ucla.edu/ (accessed on 12 December 2022)) before being used. The corresponding PDB files were downloaded and submitted to the evaluation software, which simultaneously provided six software evaluation results. Three of the results showed whether the model was available. Multiple software packages were used to determine whether the model was good or bad, and Swiss PDB Viewer was used to visualize the protein structure.

### 4.13. Gene Expression Analysis

*P. edulis* samples were stored at −80 °C, and RNA was extracted from the bamboo buds using the commonly used TRIzol method. The presence of RNA was determined by 1% agarose gel electrophoresis. The bands and quantity and purity of the total RNA were analyzed using a Bioanalyzer 2100 and RNA 6000 Nano LabChip Kit (Agilent, San Diego, CA, USA, 5067-1511). We selected RNA that met the requirements and used the EvoM-MLV Reverse Transcription Kit II (Accurate, Shenzhen, China) to synthesize complementary deoxyribose nucleic acid (cDNA) from RNA and observed the cDNA bands using agarose gel electrophoresis. The final high-quality cDNA was stored in a −20 °C freezer until quantitative real-time PCR (qRT-PCR) analysis.

Primer Premier 5 software [45] was used to design the gene-specific primers. *Glyceraldehyde 3-phosphate dehydrogenase* was used as the reference gene [46]. All gene primers used in the qRT-PCR analysis are listed in Table S1. Tissue testing for each variety was performed using three biological and three technical replicates. For PCR, 20 µL of PCR mix was prepared using 10 µL SYBR Green ProTaq HS Premix (Accurate), 8.2 µL of water, 0.8 µL of each primer, and 1 µL of cDNA template. The PCR conditions were 95 °C for 30 s followed by 40 cycles of 95 °C for 5 s and 60 °C for 30 s. PCR amplification of target genes was performed in a 96-well optical reaction plate using a real-time PCR system (Roche, Basel, Switzerland). The specificity was verified by melting curve analysis of the qRT-PCR products, and the relative expression levels of different genes were determined using the 2^−∆∆CT^ method [47].

## 5. Conclusions

We identified 81 *TLP* genes in the *P. edulis* genome. *PeTLP* genes were distributed across 24 scaffolds that were potentially derived from segment duplications during evolution. These genes were classified into three groups and ten subclasses based on the constructed intraspecific and interspecific phylogenetic trees. *PeTLP* gene promoters were found to contain multiple cis-acting elements associated with hormone and stress responses, which were consistent with the expression pattern of PeTLPs in *P. edulis* with witches’ broom disease, along with hormone, salt, or drought stress. These PeTLP expression patterns were consistent with the corresponding gene and protein structures. Overall, our findings provide a basis for further functional characterization of *PeTLP* genes and may be helpful for breeding *P. edulis* species with disease resistance or enhanced resistance to various abiotic stressors.

## Figures and Tables

**Table 1:** Detailed information of thaumatin-like proteins (TLPs) identified in *Phyllostachys edulis*.

Gene ID	Length (Bp)	MW (Da)	Isoelectric Point (Pi)	Subcellular Localization
*PH02Gene31536*	311	31,406.8	4.88	Extracellular
*PH02Gene31537*	307	31,499.0	4.41	Extracellular
*PH02Gene39670*	103	10,757.1	7.34	Extracellular
*PH02Gene37845*	196	20,776.4	6.21	Extracellular
*PH02Gene17081*	317	31,191.4	4.25	Extracellular
*PH02Gene32525*	274	28,569.2	7.90	Extracellular
*PH02Gene48120*	264	26,727.6	4.39	Extracellular
*PH02Gene08982*	80	8598.6	8.20	Extracellular
*PH02Gene31916*	275	27,777.1	4.81	Extracellular
*PH02Gene23788*	253	26,059.6	7.66	Extracellular
*PH02Gene35938*	180	18,320.7	5.08	Extracellular
*PH02Gene35939*	460	48,872.3	6.77	Plasma membrane
*PH02Gene46317*	158	17,015.2	8.35	Extracellular
*PH02Gene24694*	223	23,839.4	4.75	Extracellular
*PH02Gene38387*	263	27,608.8	7.30	Extracellular
*PH02Gene11792*	638	69,398.1	4.83	Nucleus
*PH02Gene11793*	370	38,475.5	8.63	Chloroplast
*PH02Gene18036*	207	21,692.0	4.78	Extracellular
*PH02Gene18037*	136	14,624.4	5.80	Extracellular
*PH02Gene09221*	161	16,556.4	4.12	Extracellular
*PH02Gene12336*	203	21,151.1	7.71	Extracellular
*PH02Gene12337*	184	19,016.6	4.35	Extracellular
*PH02Gene12338*	215	22,254.4	8.10	Extracellular
*PH02Gene12339*	208	21,533.5	6.48	Extracellular
*PH02Gene12340*	213	22,002.0	6.47	Extracellular
*PH02Gene12360*	214	22,082.9	6.47	Extracellular
*PH02Gene35154*	138	14,714.5	5.06	Extracellular
*PH02Gene35155*	172	17,936.2	8.08	Extracellular
*PH02Gene42065*	168	17,421.4	4.44	Extracellular
*PH02Gene45597*	170	17,323.1	4.32	Extracellular
*PH02Gene45599*	180	18,343.2	4.18	Extracellular
*PH02Gene45600*	184	18,808.0	4.95	Extracellular
*PH02Gene45601*	156	15,938.7	3.89	Extracellular
*PH02Gene45602*	178	18,157.3	7.92	Extracellular
*PH02Gene45604*	172	17,564.5	4.55	Extracellular
*PH02Gene48556*	244	25,343.4	8.12	Extracellular
*PH02Gene11255*	322	31,715.2	4.03	Extracellular
*PH02Gene15990*	341	34,912.6	4.58	Extracellular
*PH02Gene33591*	315	32,954.2	4.60	Extracellular
*PH02Gene32603*	180	19,607.2	7.34	Extracellular
*PH02Gene44064*	133	14,217.2	8.42	Extracellular
*PH02Gene14147*	320	31,644.4	5.61	Extracellular
*PH02Gene14148*	327	33,093.0	4.71	Extracellular
*PH02Gene19164*	258	26,369.3	5.59	Extracellular
*PH02Gene19402*	142	15,262.9	4.64	Extracellular
*PH02Gene19403*	167	17,698.4	4.63	Extracellular
*PH02Gene04978*	194	20,939.8	6.74	Extracellular
*PH02Gene13245*	298	32,260.4	7.27	Extracellular
*PH02Gene13252*	643	70,479.9	7.38	Extracellular
*PH02Gene13257*	607	66,805.0	7.75	Plasma membrane
*PH02Gene13259*	650	71,573.6	7.15	Plasma membrane
*PH02Gene13265*	218	22,448.2	8.28	Extracellular
*PH02Gene32549*	308	32,819.8	7.46	Extracellular
*PH02Gene14610*	106	10,444.6	8.68	Extracellular
*PH02Gene27897*	301	30,892.5	6.81	Extracellular
*PH02Gene27899*	257	26,123.8	4.31	Extracellular
*PH02Gene40419*	287	30,829.8	8.02	Extracellular
*PH02Gene43141*	313	31,722.3	4.62	Extracellular
*PH02Gene04661*	322	33,794.7	4.60	Extracellular
*PH02Gene04669*	168	17,726.9	8.36	Extracellular
*PH02Gene05114*	304	31,586.1	4.61	Extracellular
*PH02Gene05115*	314	31,623.1	4.87	Extracellular
*PH02Gene28313*	326	33,124.9	4.47	Extracellular
*PH02Gene28314*	310	32,395.1	5.31	Extracellular
*PH02Gene31888*	284	30,621.7	8.22	Extracellular
*PH02Gene05023*	334	33,810.0	5.03	Extracellular
*PH02Gene05024*	337	34,066.3	4.55	Extracellular
*PH02Gene08781*	259	26,189.2	4.39	Extracellular
*PH02Gene28355*	376	39,628.9	4.42	Extracellular
*PH02Gene07087*	319	32,854.0	4.36	Extracellular
*PH02Gene24099*	320	33,535.0	4.93	Extracellular
*PH02Gene34215*	285	30,432.4	7.90	Extracellular
*PH02Gene34852*	270	27,490.5	4.62	Extracellular
*PH02Gene50388*	104	11,751.2	8.19	Extracellular
*PH02Gene28872*	245	25,019.8	5.66	Extracellular
*PH02Gene28874*	271	27,347.4	4.63	Extracellular
*PH02Gene48321*	285	29,614.1	7.85	Extracellular
*PH02Gene32892*	270	27,520.5	4.62	Extracellular
*PH02Gene48998*	253	25,899.9	6.82	Extracellular
*PH02Gene51133*	271	27,392.4	4.83	Extracellular
*PH02Gene50762*	158	17,015.2	8.35	Extracellular

## Data Availability

The original contributions presented in the study are included in the article/Supplementary Material, and further inquiries can be directed to the corresponding authors. All the reads of witches’ broom disease bamboo tissues by RNA-Seq were deposited in National Center for Biotechnology Information (NCBI) SRA repository with accession number PRJNA980656.

## Supplementary Materials

The following supporting information can be downloaded from https://www.mdpi.com/article/10.3390/ijms241210257/s1.
