# Supplementary material for: Genome-Wide Identification and Characterization of the TLP Gene Family in Phyllostachys edulis and Association with Witches’ Broom Disease Resistance in Bamboo
Source: Int J Mol Sci. 2023 Jun 17;24(12):10257. doi: 10.3390/ijms241210257 (PMC10299529; doi:10.3390/ijms241210257)
Supplement: Supplementary file 1 [file ijms-24-10257-s001.zip › ijms-2437351-supplementary.pdf]

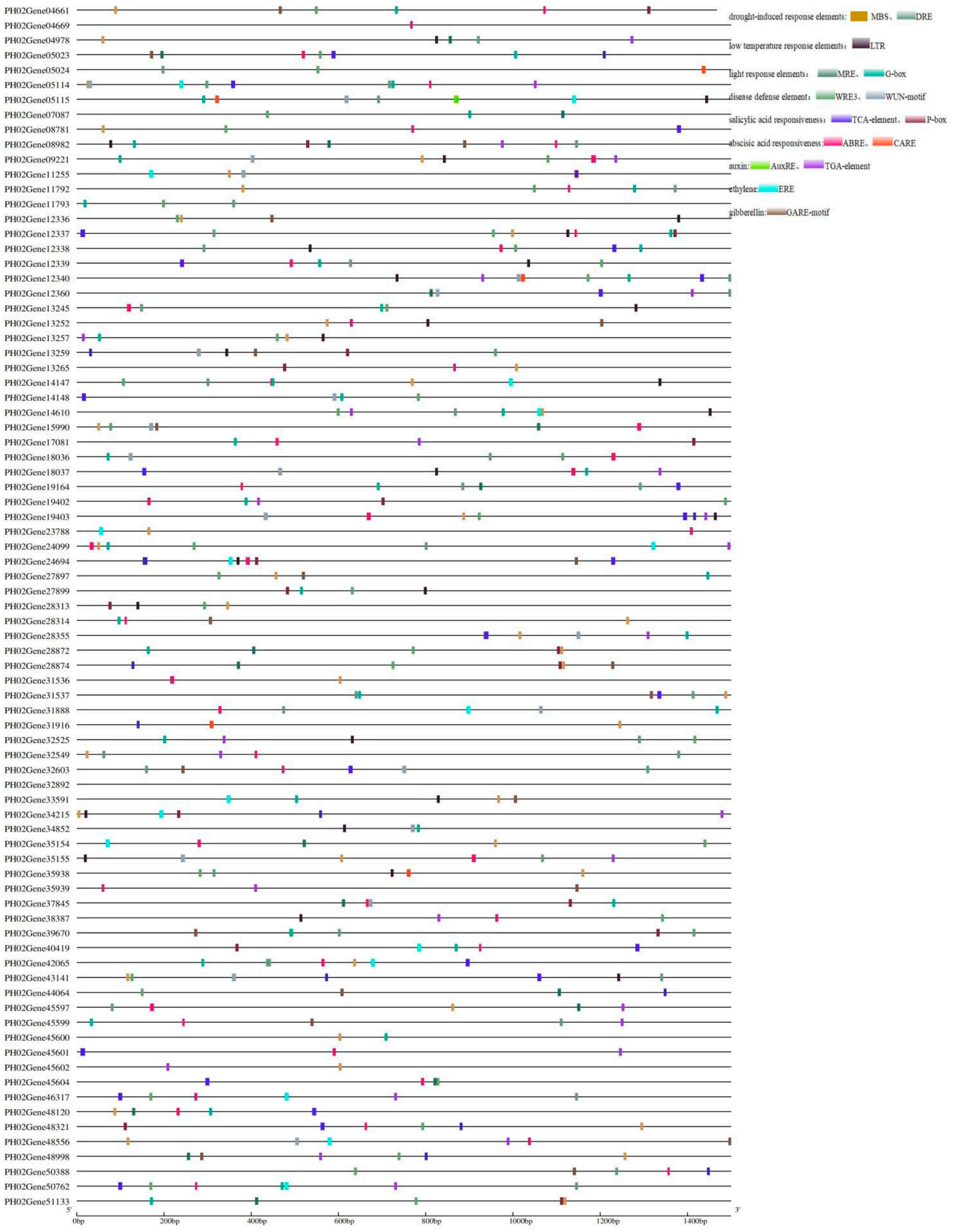

Figure S1. Predicted cis-elements of the TLP gene promoters in *P. edulis*. Different-colored rectangles represent different cis-elements.

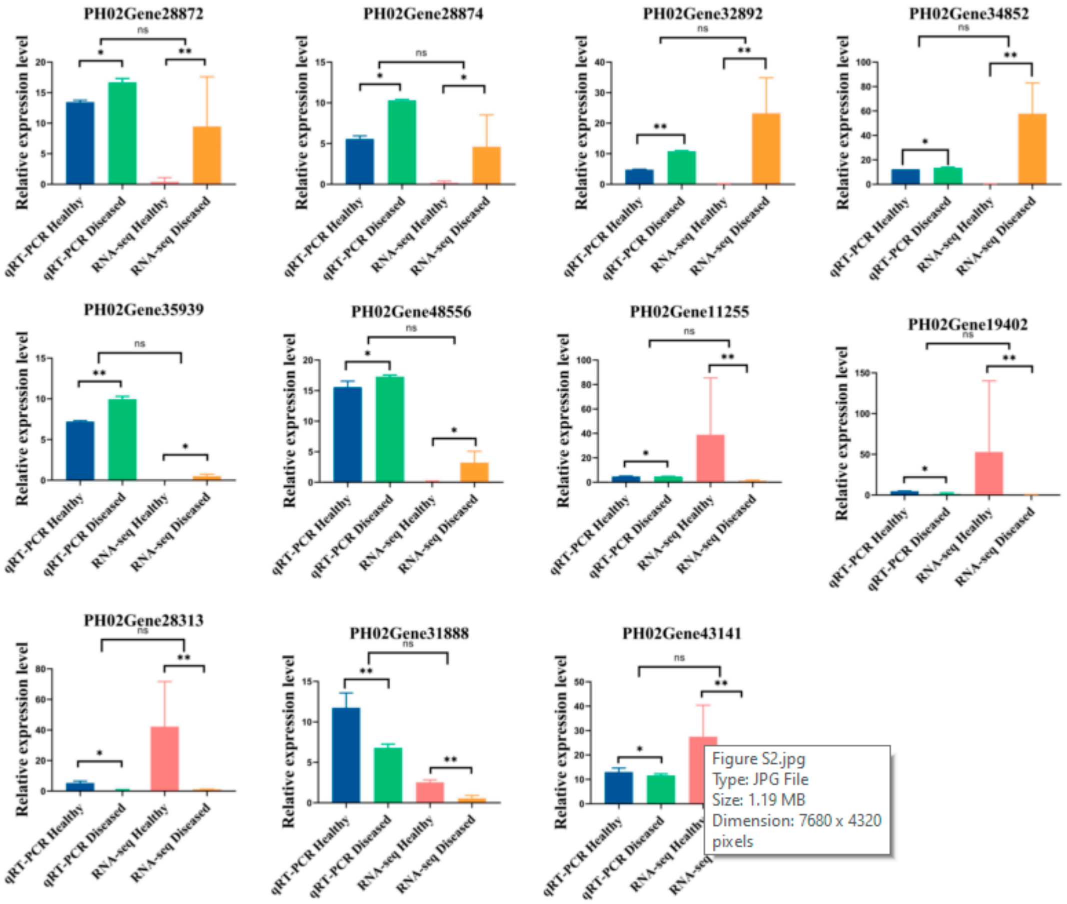

Figure S2. Comparative statistical analysis of gene expression by qRT-PCR and RNA-seq assay in healthy and diseased buds, * indicates p-value<0.05

Table S1. qRT-PCR Genes and their designed primers

Gene ID	Forward primer	Reverse primer
GADPH	CTCTTCGGCCAGAAGCCAGTCAC	TTGGCACCACCCTTCAAGTGAGC
PH02Gene28872	TGCGCGACCGACTCCAC	GTGACGTCATAGCTGGAGGC
PH02Gene28874	CGACTACTCGCAGGTGTTCA	GAAGGTGACGTCATAGCTGGA
PH02Gene32892	TACTGCTGCACCGGTGATT	GAAGGTGACGTCATAGCTGGA
PH02Gene34852	GTGTGGGTGGGCTCGCTCAA	GAAGTCCTGGCCTCCGCTGC
PH02Gene35939	AATTACGCTGTCCCTCGCCG	AGGTTGTGGAGGGTGAAGGTA
PH02Gene48556	CATCAACTTGCCCGCCG	AGTCCAGGTCCCTCCCC
PH02Gene11255	CAGCTACGCATACGACGACT	GCAGAAGGTGATGGCGTAGT
PH02Gene19402	CGTACAGCTACCCCAAGGAC	ACGATCTGGTAGTTGGTGCC
PH02Gene28313	CCTACAGCTACGCCTACGAC	CTGGGGCAGAAGGTGATGTC
PH02Gene31888	CCCACCACCTACTCCAAGAA	AGCACAAGCATCAGGGTAGG
PH02Gene43141	CAAGTTCACATGCGTCACCG	GACCAGGCTGACGTCGTAAA
